# Replication Timing of Human Telomeres Is Chromosome Arm–Specific, Influenced by Subtelomeric Structures and Connected to Nuclear Localization

**DOI:** 10.1371/journal.pgen.1000920

**Published:** 2010-04-22

**Authors:** Nausica Arnoult, Caroline Schluth-Bolard, Anne Letessier, Irena Drascovic, Rachida Bouarich-Bourimi, Judith Campisi, Sahn-ho Kim, Amina Boussouar, Alexandre Ottaviani, Frédérique Magdinier, Eric Gilson, Arturo Londoño-Vallejo

**Affiliations:** 1 Telomeres and Cancer Laboratory, Institut Curie, CNRS, UPMC University Paris 06, Paris, France; 2 Epigenetics and Telomere Regulation, CNRS ENS UCBL IFR128, Ecole Normale Supérieure de Lyon, Lyon, France; 3 Functional Organization and Plasticity of Mammalian Genomes, Institut Curie, UPMC University Paris 06, Paris, France; 4 Lawrence Berkeley Laboratory, Berkeley, California, United States of America; 5 Buck Institute for Age Research, Novato, California, United States of America; Brandeis University, United States of America

## Abstract

The mechanisms governing telomere replication in humans are still poorly understood. To fill this gap, we investigated the timing of replication of single telomeres in human cells. Using in situ hybridization techniques, we have found that specific telomeres have preferential time windows for replication during the S-phase and that these intervals do not depend upon telomere length and are largely conserved between homologous chromosomes and between individuals, even in the presence of large subtelomeric segmental polymorphisms. Importantly, we show that one copy of the 3.3 kb macrosatellite repeat D4Z4, present in the subtelomeric region of the late replicating 4q35 telomere, is sufficient to confer both a more peripheral localization and a later-replicating property to a de novo formed telomere. Also, the presence of β-satellite repeats next to a newly created telomere is sufficient to delay its replication timing. Remarkably, several native, non-D4Z4–associated, late-replicating telomeres show a preferential localization toward the nuclear periphery, while several early-replicating telomeres are associated with the inner nuclear volume. We propose that, in humans, chromosome arm–specific subtelomeric sequences may influence both the spatial distribution of telomeres in the nucleus and their replication timing.

## Author Summary

Functional telomeres are essential for genome stability. While replication of telomeres has been extensively studied in model organisms such as the baker's yeast, little is known about the mechanisms that govern the replication of human telomeres. In this study, we have determined the timing of replication of telomeres of individual human chromosomes and its association with potential modulating factors such as particular subtelomeric sequences, the presence of heterochromatic regions, and nuclear localization. We have found that native telomeres associated with D4Z4 sequences—a macrosatellite naturally located in the subtelomeric regions of 4q, 10q, and acrocentric chromosome extremities—replicate later than others. We also present descriptive and experimental evidence indicating that nuclear localization influences the timing of telomere replication. These results contribute to our understanding of telomere metabolism in humans.

## Introduction

Cell proliferation potential is a critical attribute that directly influences embryogenesis, development and growth. For instance, insufficient proliferation capacity compromises organogenesis, tissue regeneration and repair, while unrestrained cell proliferation promotes cancer progression [1]. The human chromosome structures that have been most directly linked to cell proliferation control are telomeres [2].

Telomeres are specialized nucleoprotein complexes found at the ends of linear chromosomes. In vertebrates, they consist primarily of thousands of double stranded hexameric repeats (5′-T_2_AG_3_-3′) that end in a 3′ G-rich protruding single strand. The double strand region is directly bound by specific telomeric factors (TRF1 and TRF2), while the 3′ overhang is bound by POT1. Interactions of these proteins with three other telomeric proteins (TIN2, TPP1 and RAP1) constitute the shelterin/telosome complex, which is required for telomere function [3], [4].

Telomeres protect chromosome ends from degradation and fusion. They ensure the complete replication of chromosomes by creating a buffer of expendable sequences. Because of both the end replication problem, following which conventional DNA polymerases cannot completely replicate the ends of linear molecules [5] and the post-replication processing required to form a new functional telomere [6], telomeres shorten with every genome replication cycle. In the absence of a mechanism to add telomere repeats to the 3′ end, telomeres shorten with cell division until they reach a critical length, incompatible with proper telomere function [2]. A checkpoint signal is then triggered and cells enter senescence. Cell proliferation capacity is thus determined by initial telomere length and telomere shortening kinetics [2]. The latter is highly variable among human cell lines and ranges from 30 to 300 bp/cell division [7]–[9]. In vivo, telomere shortening in haematopoietic tissues has been estimated about 25–35 bp/year, although this pace is accelerated during the first years of life and also under stress or pathological conditions [10]–[16].

Telomerase, the dedicated reverse transcriptase that adds telomeric repeats *de novo* to the 3′ end, is highly active during development and its activity persists in stem cell compartments, where it ensures the cell replication potential of highly proliferative tissues [17]–[19]. However, as suggested by the telomere shortening that occurs with aging, the levels of telomerase are limiting [18], [20]. Also, mutations that prevent full telomerase activity accelerate telomere shortening and cause the premature appearance of aging phenotypes [21]–[23]. Accelerated telomere shortening might also result from difficulties during telomere replication. For instance, it has been shown that mutations in the gene coding for the WRN exonuclease/helicase compromises the replication and integrity of the telomere G-rich strand [24], [25]. Telomere replication defects may thus contribute to the aging phenotypes observed in Werner syndrome patients [26], [27].

Very little is known regarding the control of telomere replication in human cells [28]. The bulk of human telomere sequences replicate all through S-phase [29], [30]. This is seemingly different from what is observed in budding yeast where telomeres replicate in concert late in S phase [31], [32], although it is not known whether replication timing for individual human telomeres is spatially or temporally controlled. In a recent study, it was shown that telomeres in the muntjac deer display defined timings of replication and that telomeres on long and short arms replicate asynchronously [33]. This finding suggests that the firing of subtelomeric origins of replication in this species is subjected to chromosome arm-specific control mechanisms.

We have used the ReDFISH-based approach, described previously by Zou et al for the muntjac [33], to determine the timing of replication of individual telomeres in human cells. Our observations indicate that both chromosome arm-specific subtelomeric composition and nuclear localization influence the timing of telomere replication in humans.

## Results

### Single human telomeres replicate at preferential moments during the S-phase

**Figure 1:**
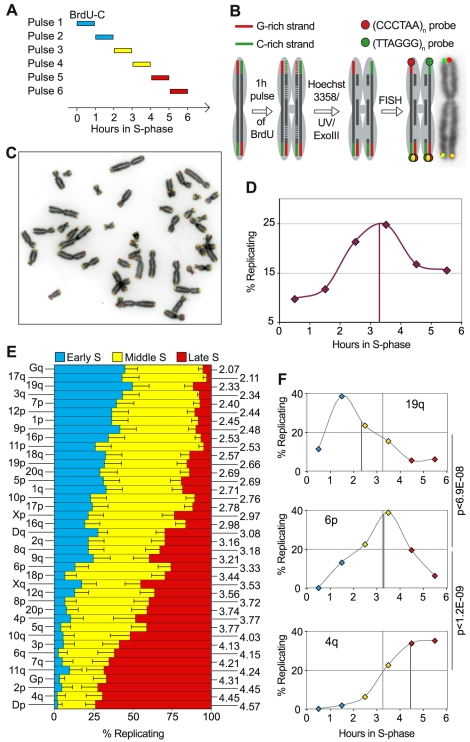
Human telomeres replicate within limited and chromosome arm-specific time windows. (A) The ReDFISH approach is based on the labeling of synchronized cells with BrdU & BrdC in pulses of 1 hour covering the entire S phase. Timing of entry in S-phase after aphidicolin release was ascertained by FACS analysis (not shown). (B) BrdU-C is incorporated in sister chromatid regions that replicate during the pulse. The CO-FISH procedure destroys all base-substituted strands, thus sister telomeres that replicated during the pulse are converted into single strands and therefore recognized exclusively by either C-rich or G-rich specific probes (red and green signals, respectively). Telomeres that are not detargeted by the procedure yield mixed hybridizations (yellow signals). (C) IMR90 metaphase spread after ReDFISH. (D) Overall replication timing of telomeres in IMR90 cells. Diamonds indicate the percentage of detargeted telomeres observed during each pulse of BrdU-C. FACS analysis indicated that the S-phase was finished little after 6 hours (not shown). The vertical line indicates the mean replication timing (mrt) for all telomeres in IMR90 cells (i.e.: 3.12h). (E) Replication timing of individual telomeres in IMR90 cells. The percentage of replicating telomeres for every chromosome arm detected during pulses 1+2 (early S), 3+4 (middle S) and 5+6 (late S) is represented in horizontal bars. The total sum of partial percentages is normalized to 100% with horizontal lines inside bars representing confidence intervals (c.i.) (α = 0.05). For the sake of simplicity, only upper limits for early S and lower limits for late S are represented. Chromosome arms are listed (left) from the lowest to the highest mean replication timing (right). Acrocentric chromosomes are grouped according to the following convention: group D for chromosomes 13, 14 and 15 and group G for chromosomes 21 and 22. (F) Examples of early (19q), middle (6p) and late (4q) replicating telomeres. The vertical black line indicates the mrt for these telomeres, while the vertical grey line indicates the mrt for all telomeres.

We used human primary fetal lung fibroblasts (IMR90) and the ReDFISH approach, which is a modified version of the CO-FISH technique [34] (Figure 1A–1C), to characterize the timing of replication of individual telomeres. At least 30 metaphases were analyzed for each hour of a BrdU/C pulse to determine the percentage of individual telomeres being replicated and to calculate the mean replication timing (mrt) for each chromosome arm.

Global analysis showed that telomere replication takes place during the whole S-phase with a peak (about ¼ of all telomeres) during the fourth hour after S-phase initiation (mrt: 3.27; Figure 1D). This kinetics of bulk telomere replication has already been observed using density-labeling methods [35] or BrdU-based detection [30], [36]. However, our results indicate that single telomeres replicate in less than one hour since 1 hour BrdU pulses are sufficient to reveal perfectly detargeted sister telomeres (i.e. telomeres on homologous sister chromatids that are exclusively recognized by either G-rich or C-rich specific probes, Figure 1C). Moreover, telomeres located at specific chromosome ends tend to preferentially replicate during a defined window of the S-phase, with some telomeres replicating rather early and others replicating late (Figure 1E). For instance, 50% of telomeres on the 19q arm replicate during the first two hours (mrt: 2.33, Figure 1F), whereas around 70% of telomeres on the 4q arm replicate during the last two hours (mrt: 4.45, Figure 1F). Both mean replication timings are significantly different from the mean replication timing of 6p, a mid-S replicating telomere (mrt: 3.33, Fisher exact tests: 19q vs 6p, p = 6.9×10^−8^, 4q vs 6p, p = 1.2×10^−9^ and 4q vs 19q, p = 2.2×10^−16^. Significance threshold: p<0.0025). As observed in muntjac cells [33], telomeres on short and long arms of the same chromosomes show no coordinated replication.

There is no obvious correlation between the reported replication pattern of the last R/G (R = reverse, G = giemsa) cytogenetic bands on each chromosome arm, revealed also by incorporation of BrdU [37]–[39], and the pattern of replication for single telomeres observed here. There is a weak, albeit not significant, correlation of single telomere replication timings and the mean replication timings reported for the most distal chromosome-specific regions included in BAC (bacterial artificial chromosome) arrays (Figure S1) [40] suggesting some synchronicity between telomeres and distal subtelomeric regions (Spearman's rank correlation test: p = 0.093, significance threshold p<0.015).

### Length does not impact replication timing of human telomeres

**Figure 2:**
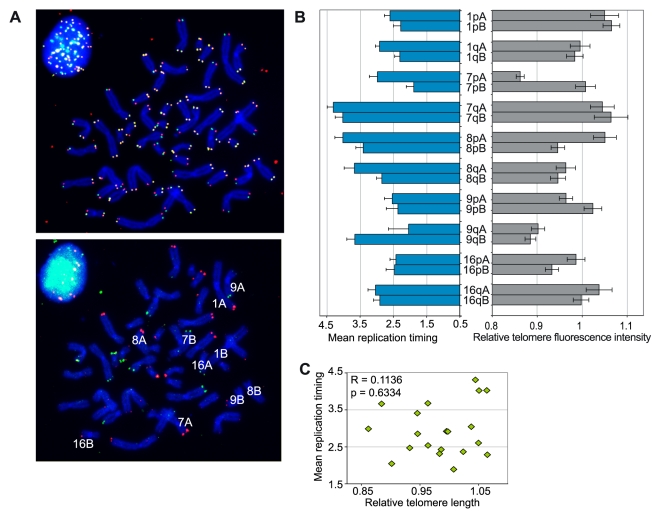
No apparent relationship between replication timing of single telomeres and their length. (A) Example of an IMR90 metaphase treated sequentially for ReDFISH (top) and for subtelomeric FISH (bottom) to distinguish between homologous chromosomes. In IMR90 cells, the subtelomeric probes used (f7501 and ICRF10, revealed in green and red colors, respectively) allow us to distinguish between the homologs of chromosomes 1, 7, 8, 9, and 16. (B) Mean replication timings and relative telomere lengths (measured by Q-FISH) for each allele are plotted side by side. (C) Absence of correlation between mean replication timing and relative telomere lengths.

To understand which factors regulate the replication timing of individual telomeres, we examined the impact of telomere length and telomerase expression. Indeed, recent work in budding yeast suggests that telomere length could influence the timing of replication. Particularly, a shortened telomere tends to replicate earlier [41] while the bulk of telomeres replicate rather late [42]. Using telomere Q-FISH followed by subtelomeric FISH as described previously [9], we measured relative telomere fluorescence intensities (which indicate telomere length relative to the mean telomere length of the cell) specifically associated with polymorphic chromosome arms. We used the same subtelomeric FISH approach after ReDFISH to determine the replication timing of telomeres of the same chromosome pairs (Figure 2A). Although significant telomere length differences exist between some alleles, their telomeres replicated with similar timings (7p, 8p or 16p) (Figure 2B). Also, some allelic telomeres, like those on 9q, showed differences in mean replication timing although no difference in telomere length was observed (Figure 2B). A global comparison of telomere lengths and mean replication timings through a correlation analysis confirmed that no relationship exists between both variables (Figure 2C).

**Figure 3:**
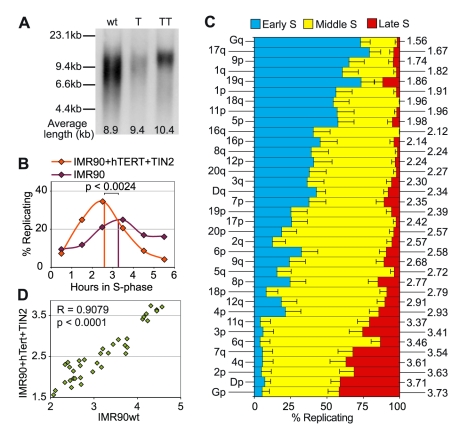
Telomere elongation by telomerase does not impact on the replication timing of single telomeres. (A) Expression of telomerase activity increases and homogenizes telomere lengths. Telomere restriction fragment analysis of IMR90 (wt), IMR90+hTERT (T) and IMR90+hTERT+TIN2 (TT) was performed by Southern blotting using RsaI/HinfI-digested DNA and probing with a ^32^P labelled telomeric oligonucleotide (CCCTAA4). Mean telomere length was calculated using Telometrics, which showed that telomere length increases in cells bearing telomerase. (B) Overall replication timing of telomeres in IMR90+TT cells. Diamonds indicate the percentage of detargeted telomeres observed during each pulse of BrdU-C. Start of S-phase was ascertained by FACS analysis, which also indicated replication was completed in 5.5 hours (not shown). For comparison, the replication timing of telomeres in the parental IMR90 cells is also shown. Overall telomere replication in IMR90+TT cells appears to occur slightly earlier than in parental cells (mrt = 2.61 indicated by the orange vertical line). (C) Pattern of replication of single telomeres in IMR90+TT cells during the S-phase. The mrt for each telomere is indicated on the right. (D) A significant correlation of telomere-specific mean replication timings exists between IMR90 cells and its immortalized derivative IMR90+TT cell line with longer telomeres, indicating that the relative order of telomere replication was not significantly altered.

To further corroborate this observation, we examined the replication profile in IMR90 cells expressing the catalytic subunit of human telomerase (hTERT). hTERT is limiting for telomerase activity in most human fibroblasts and is often sufficient to increase their replication potential [43]. However, some cells spontaneously increase the expression of p16INK4a by mechanisms that are unknown and such cells senesce even in the presence of telomerase activity. IMR90+hTERT cells fall into this category [44], preventing us from obtaining enough analyzable material for our studies. We therefore expressed in these cells TIN2, another telomeric factor [45]. TIN2, through its interaction with TRF1, exerts a negative control on telomere length. In our cells, however, telomeres were stabilized above 10 kb with individual telomere lengths being largely homogenized, as expected for a cell line expressing hTERT alone (Figure 3A) [9]. IMR90+hTERT+TIN2 cells grew vigorously, allowing us to perform the same study in cells that had longer and much more homogeneous telomeres than primary cells. The length of S-phase, as indicated by FACS analysis, is somewhat shorter in telomerized IMR90 cells, since it lasts 5.5 hours instead of a little more than 6 hours in the parental cell line (not shown). Concurrently, telomere replication peaks earlier (mrt: 2.61) and very few telomeres are seen replicating during the last pulse (Figure 3B). Remarkably, however, the relative timings of replication for single telomeres in this cell line were very similar to the one observed in unmodified cells (Figure 3C). A statistical analysis (Figure 3D) showed a highly significant positive correlation (Spearman's rank correlation test: p<0.0001, significance threshold p<0.01), indicating little variation in the relative timings of telomere replication between both cell lines. These results strengthen the conclusion that telomere length has no visible impact on telomere replication timing in humans. Interestingly, the mean replication timings for single telomeres in telomerized IMR90 cells appear to be significantly correlated to the mean replication timings of the most distal chromosome-specific sequences reported by Woodfine et al [40] (Spearman's rank correlation test: p = 0.0062) (Figure S1).

### The pattern of individual telomere replication is conserved between homologous chromosomes and between cells from different individuals

Subtelomeric regions show extensive segmental polymorphisms, which can reach several hundreds of kilobases [46]–[48] and could directly impact replication origin firing and/or replication fork speed. In the experiment to determine the relative lengths of allelic telomeres, we used subtelomeric probes recognizing segments (around 30–40 kilobases long) that are present or absent at chromosome extremities and are located very close to the telomere tract (10 to 20 kb) [46], [48], [49]. As shown in Figure 2B, we found that extremities corresponding to allelic locations but carrying segmental polymorphisms (alleles labeled A and B on 1q, 7p, 8p, 9p and 16q in Figure 2A) tend to replicate during the same time window, like do homologous sequences elsewhere in the genome [50]. On the other hand, telomeres on different chromosome pairs but associated with subtelomeric regions of similar segmental composition (compare for instance 1pA and 7pA to 8pA and 9qB) may have different timings of replication. This indicates that segmental polymorphisms do not account for the differential replication timing of individual telomeres.

**Figure 4:**
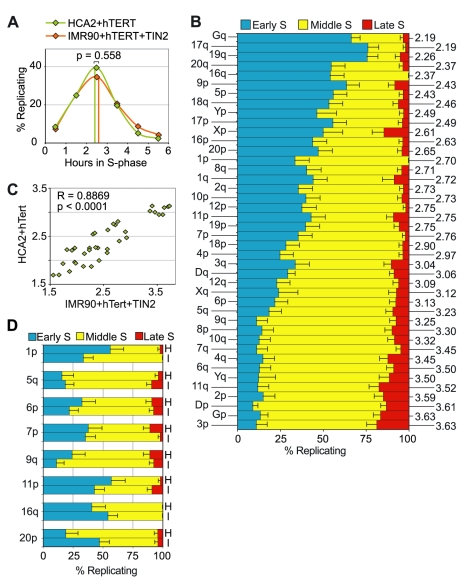
The replication timing of single telomeres is conserved among individuals. (A) The overall telomere replication profile in HCA2+T cells (mean replication timing: 2.45h, green vertical line) is very similar to the one observed in IMR90+TT cells. (B) Pattern of replication of single telomeres in HCA2+T cells during the S-phase. The mrt for each telomere is indicated on the right. (C) A high correlation is observed between telomere-specific mrt of HCA2+T and IMR90+TT cells, indicating that the relative order of telomere replication is conserved. (D) Specifically, no major differences are detected between the profiles of telomere replication of chromosome arms known to carry subtelomeric segmental variations in HCA2+T (H) and IMR90+TT (I).

To determine whether telomeres with identical chromosome positions in the genome tend to replicate at similar times in different individuals, we studied the telomere replication pattern in the foreskin fibroblast cell line HCA2, which expresses an exogenous copy of hTERT and therefore replicates indefinitely, like the IMR90+hTERT+TIN2 cells. In fact, both the length of the S-phase and the global timing of telomere replication are indistinguishable between both cell lines (Figure 4A). Even more remarkably, the ranking of mean replication timings for individual telomeres was very similar (Figure 4B), as indicated again by a statistically significant positive correlation coefficient (Figure 4C). Also striking is the observation that chromosome extremities potentially carrying extended subtelomeric segmental variations in both cell lines harbor similar replication timings (Figure 4D), strengthening the idea that these genetic polymorphisms do not have a major effect on telomere replication timing. Again, the mean replication timings for single telomeres in telomerized HACA2 cells appear also to be correlated to the mean replication timings of the most distal chromosome-specific sequences (Figure S1).

Interestingly, the most conspicuous, albeit limited, variations in replication timing between IMR90 and HCA2 cells concern telomeres that replicate in the first two thirds of the S-phase, while less variation is apparent amongst telomeres that replicate later (Figure 4C). Since the incidence of subtelomeric polymorphisms is equally distributed among early and late replicating telomeres, this observation suggests that differences in replication timing because of genetic variations might be more easily superseded by factors causing telomeres to replicate late in the S-phase. We therefore conducted a closer examination of late replicating telomeres.

### Telomeres on the long arm of sex chromosomes are late replicated

It has been suggested that transcriptionally inactive heterochromatic regions tend to replicate during the second part of the S-phase [51]. Also, in females, both X chromosomes display a different replication pattern according to their heterochromatic state. Active X chromosomes behave like autosomal chromosomes, bearing early and late replicating bands, while inactive X (Xi) shows a pattern of late replication that generally encompasses the entire chromosome [38]. Our analysis of telomere replication by ReDFISH revealed that, on the X chromosomes of IMR90 cells, telomeres on the short arm replicate during the middle of S-phase, rather synchronously as expected for homologous chromosomes. Replication of telomeres on the long arm presented a bimodal distribution with one peak of replication in the middle of S-phase and another peak at the end of that phase (Figure S2). The late profile of BrdU incorporation observed all along the chromosome that also presented a late replicating telomere suggested that this chromosome is Xi (not shown). However, since both features depend on the same phenomenon (BrdU incorporation during replication), confirmation that this is a bona fide Xi requires a replication-independent criterion. Unfortunately, detection of Xi-specific heterochromatic marks (such as particular histone modifications) was precluded by the type of chromosome fixation (ethanol/acetic acid) used in the ReDFISH approach. In the male HCA2+T cells, the Yp telomere replicated early in S-phase while the Yq telomere displayed a much later replication pattern (Figure S2) (mrt: 2.49 and 3.50, respectively, Fisher exact test: p = 9.9×10^−8^), suggesting that the replication timing of this telomere might be influenced by the constitutive heterochromatic region found on the Yq arm. On the other hand, although the Xq telomere shows a peak of replication coincident with the Xp telomere, their calculated mrts are significantly different (2.61 and 3.12, respectively, Fisher exact test: p = 8.7×10^−6^) (Figure S2) indicating that Xq replicates later than Xp in this male fibroblast cell line. On the other hand, the comparison between the mean replication timings of Xq and Yq telomeres fails to show a significant difference confirming that both telomeres have a late replicating pattern.

### Telomeres on chromosome ends carrying satellite-like repeats replicate late

**Figure 5:**
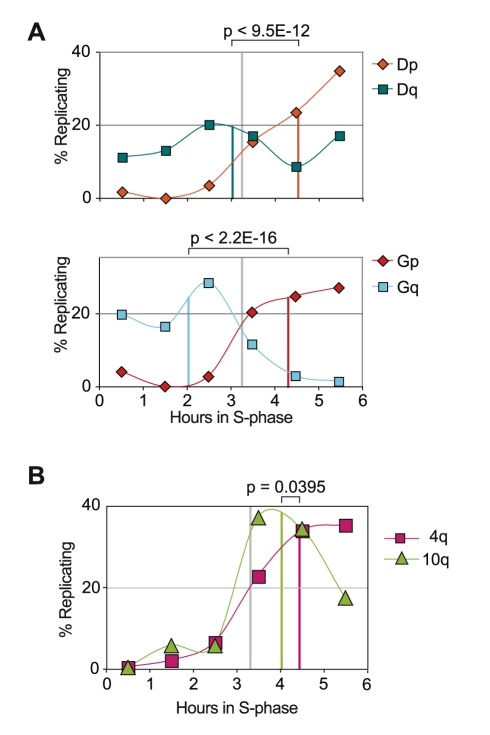
Telomeres associated with satellite-like subtelomeric repeats are late replicating. (A) Late telomere replication of the short arms of acrocentric chromosomes in groups D (13, 14, and 15) and G (21 and 22), compared to the respective q arms, in IMR90 cells. Similar replication patterns are detected in all cell lines examined in our study (Figure 3C, Figure 4B, and Figure 7C). (B) Late replication profile of telomeres located on 4q and 10q chromosome ends in IMR90 cells. 4q and 10q are also late replicating in HCA2+T cells (Figure 4B). Colored vertical lines indicate the mean replication score for each telomere. No statistically significant differences are observed between 4q and 10q mrts (significance threshold p<0.0025).

Amongst the telomeres that consistently replicated late during S-phase in all cells examined are those located on the short arms of acrocentric chromosomes (Figure 1E, Figure 3C, Figure 4B, and Figure 5A). Together with rDNA clusters, acrocentric regions carry both β-satellite sequences and D4Z4 repeats [52]. These two last kinds of repeats are also found at two other extremities, 4qter and 10qter. As shown in Figure 5B, both 4q and 10 replicate late in IMR90, a behavior also observed in IMR90+TT and HCA2+T cells (Figure 3C and Figure 4B). These observations suggest that the presence of satellite-like repeats at subtelomeric positions may influence the timing of telomere replication. However, while telomeres on acrocentric short arms have been detected as associated with the nucleolus [53], both 4qter and, although much less consistently, 10qter have been reported as being associated with the nuclear periphery [54]–[56], suggesting that nuclear localization could also influence the timing of telomere replication. We therefore tested both the replication timing and nuclear localization of newly created telomeres carrying a defined subtelomeric composition.

### A more peripheral nuclear localization is associated with a later replication timing

**Figure 6:**
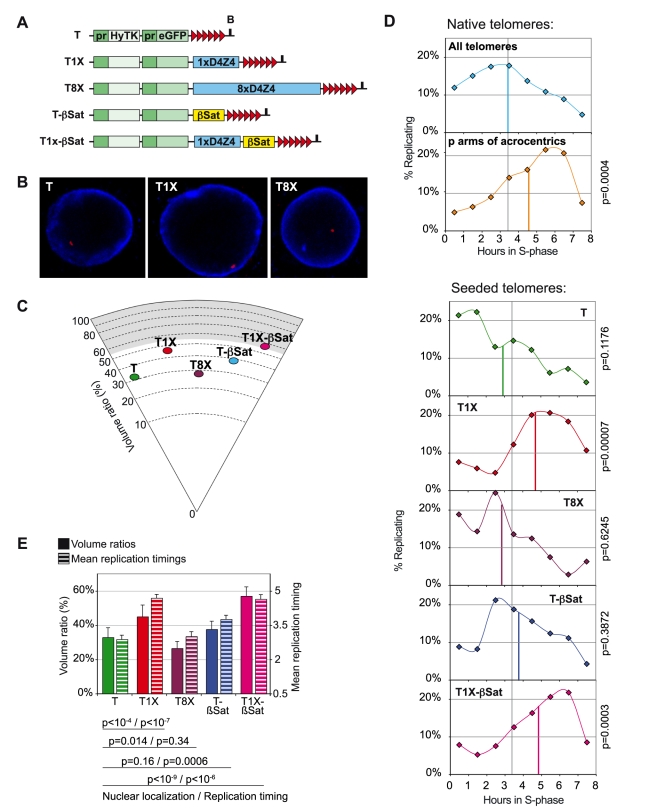
The replication timing of seeded telomeres correlates with their nuclear localization. (A) Constructs used for random telomere seeding in C33A cells and carrying specific subtelomeric elements events as described in [56] and [58]. T: no subtelomeric sequences; T1X: carries a 3.3kb D4Z4 element [56]; T8X: carries 8xD4Z4 elements in tandem [56]; T-Sat: carries a 1.4kb subtelomeric fragment in 4q35, distal to D4Z4 sequences and spanning 4 β-satellite repeats (Boussouar et al., manuscript in preparation); T1X-βSat: carries a D4Z4 element next to the native subtelomeric fragment of 4q spanning 4 β-satellite repeats [56]. (B) Nuclear localization by immuno-3D of randomly seeded telomeres in the C33A cell line. Shown are examples of FISH signals (in red) obtained for control seeded telomeres (T) and telomeres associated with 1 copy (T1X) or multiple copies (T8X) of D4Z4. Lamin B, the reference for the nuclear periphery, is revealed in blue. (C) Constructs carrying only one D4Z4 repeat show a more peripheral localization in immuno-3D. Represented is the mean position of these telomeres (n = 50) in a circle section indicating percentages of volume ratios, calculated as described [56]. The gray shadow indicates the value for mean volume ratio obtained for lamin B. (D) Telomere replication timings of native and seeded telomeres in C33A cells. mrt for each telomere is indicated by colored vertical lines. The grey line indicates the mrt (3.5) for all telomeres in C33T1X -whose overall telomere replication profile is shown on the top panel. The replication profile of telomeres on the short arms of acrocentric chromosomes is also shown (mean replication timing: 4.57). The significance values obtained by comparing mrt for seeded telomeres and the mean replication timing of all native telomeres in the C33A cells are also shown. Only T1X and T1X-βSat (mean replication timings 4.7 and 4.65, respectively) replicate significantly later. (E) Mean volume ratios for nuclear localization (fully colored bars) and mean replication timings (stripy bars) of single telomeres are plotted together. Vertical lines on top of the bars indicate the standard errors for both measurements. The significance values of comparisons taking both sets of data between the control telomeres, on one hand, and the test telomeres, on the other, are given (first, between nuclear localization data; second, between replication timing data). T1X, T-βSat and T1X-βSat replicate significantly later than control telomeres, while only T1X and T1X-βSat are significantly more peripheral than the control. Significance threshold: p<0.003.

To address the specific contributions of subtelomeric elements with regard to nuclear localization and telomere replication, we artificially tagged telomeres in C33A human cells with DNA molecules that carry either multiple D4Z4 repeats, a single D4Z4 repeat, 4 β-satellite repeats or both (Figure 6A). Upon chromosome integration, such constructs lead to non-targeted (random) de novo telomere formation [57]. We have previously shown that in this cell line, polyclonal populations of stably transfected cells are representative of pools of independent clones of tagged telomeres allowing us to perform analyses on populations [56], [58], [59]. Also, the presence of particular subtelomeric sequences does not bias the chromosome integration sites of the seeding constructs [56], [58].

As shown in a previous study [56], [58], a single D4Z4 repeat, alone or inserted together with β-satellite repeats, confers to a chromosome extremity a more peripheral position within the nucleus while multiple copies of D4Z4 repeats (Figure 6B and 6C), or several β-satellite repeats (Figure 6C) alone, do not. We then determined the replication timing of all types of telomere seeded extremities and found that those carrying only one D4Z4 repeat (and bearing a more peripheral localization in the nucleus) replicate later than the others, suggesting that nuclear localization influences telomere replication timing (Figure 6D and 6E). On the other hand, the mean replication timing of telomeres connected to β-satellite sequences alone is significantly higher than the mean replication timing of control telomeres (p = 0.0006, significance threshold p<0.003). This effect is independent of nuclear localization, thus allowing the conclusion that β-satellite sequences by themselves cause a delay in telomere replication.

### Native, non-D4Z4–associated, late-replicating telomeres are localized at the nuclear periphery

**Figure 7:**
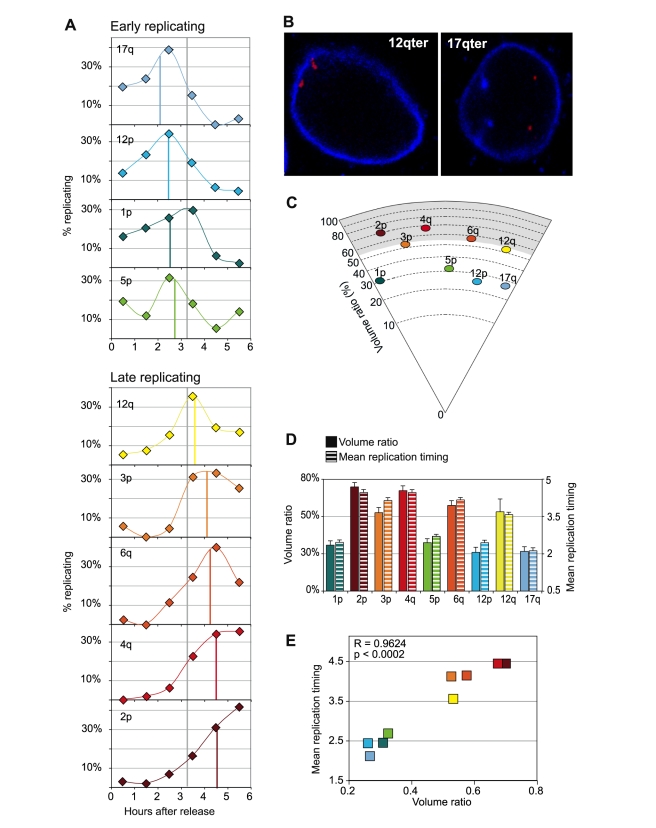
Relationship between peripheral nuclear localization and late replication timing of telomeres. (A) The replication profiles of early replicating telomeres 17q, 12p, 1p, and 5p and late replicating telomeres 12q, 3p, 6q, 4q, and 2p (mean replication timings indicated by colored vertical lines) are represented. The vertical grey lines indicate the median replication score for all telomeres. (B) Examples of nuclear localization analyses by immuno-3D for 12qter (BAC RP11-349K16) and 17qter (BAC RP11-637C24). Hybridizations are revealed in red while lamin B, the reference for nuclear periphery, is revealed in blue. (C) These analyses indicate that the late replicating telomeres 2p, 3p, 4q, 6q, and 12q are more peripheral than the early replicating telomeres 1p, 5p, 12p, and 17q. Represented is the mean position of these telomeres (n = 50) in a circle sector indicating percentages of volume ratios, calculated as described in [56]. The gray shadow indicates the value for volume ratio obtained for lamin B. (D) The mean volume ratios for nuclear localization (fully colored bars) and the mean replication timings (stripy bars) for single telomeres are plotted. Error bars indicate the standard error of the mean. (E) There is a significant correlation between the mean replication timing of single telomeres and their mean volume ratio.

Given the above results, we examined by immuno-FISH and 3D imaging the nuclear localization of native chromosome ends carrying telomeres that replicated either early or late in IMR90 primary cells. Our results, illustrated in Figure 7, indicate that the late replicating extremities 2pter, 3pter, 4qter, 6qter and 12qter have a clear tendency to localize at or near the nuclear periphery, whereas the early replicating extremities 1p, 5p, 12p and 17q are found in the inner part of the nuclear volume (Figure 7). Furthermore, a correlation analysis (p<0.0002) clearly indicates that there is a direct relationship between the mean replication timing of a telomere and its mean volume ratio determined by immuno-3D (Figure 7E). Together, our data suggest, for the first time, a strong association between telomere replication timing and nuclear localization.

## Discussion

We characterized the replication timing of single telomeres in normal diploid human cells, either primary or immortalized by ectopic expression of telomerase. In agreement with previous studies [29], [30], we found that bulk telomeres replicate throughout the S-phase. Our results further indicate that single telomeres on specific chromosome ends tend to replicate during defined times in the S-phase and that this timing is conserved between homologs and among individuals. Contrary to findings in the budding yeast [31], [32], telomere length does not have a major impact on telomere replication timing. However, given the length of the S-phase and the inherent imprecision of the methodology used, it remains possible that subtle influences introduced by the length of telomeres and/or the presence of telomerase activity may have been overlooked.

Occasionally, small differences were detected, both between homologs and among individuals, which could be explained by variations either in the DNA sequence or the epigenetic status of these extremities. Nevertheless, our study also shows that the segmental polymorphisms (which may span up to hundreds of kilobases) occurring very close to telomeres [47], [48], [60], [61] do not exert a major influence in the replication timing of allelic telomeres. The subtelomeric duplications f7501 and ICRF10 revealed in these experiments are present in about 15 chromosome extremities, a dozen of which are potentially polymorphic. These sequences are located quite close to the telomere tract and their presence or absence indirectly indicate the presence or absence of other subtelomeric segments with which they are commonly associated. For instance, in the cell line we examined (IMR90), the presence or absence of ICRF10 on chromosome 8p (Figure 2A) implies the presence or absence, respectively, of at least three other (more proximal) segments in that extremity (see [48]). This signifies that both alleles differ from each other in their subtelomeric region by at least 120kb [46]. Whether or not this distance is sufficient to introduce a difference in the replication timing for both telomeres (either by delaying the arrival of the replication fork to the telomere or by introducing a new origin of replication) remains to be explored. Nevertheless our experiments do suggest that such polymorphisms may occur without inducing major differences in telomere replication timing. On the other hand, some of the observable differences affect chromosome extremities without (known) segmental variation at subtelomeres, suggesting that other factors are at play.

Previous studies on the replication timing of specific subtelomeric regions (for instance 22q [62] and 16p [63]) suggested that particular telomeres replicate late. The present study did not detect such trend for these particular ends in the cell lines examined. Moreover, these two telomeres are among the earliest to replicate in S phase. The aforementioned studies used subtelomeric probes and interphase nuclei FISH to follow the duplication of signals during S phase progression. However, duplication of signals depends not only on replication of that particular segment but also on the resolution of sister chromatids. This step seemingly follows a different pathway at telomeres [64], which might explain why telomeres placed nearby other sequences may influence (i.e.: delay) the appearance of distinct FISH foci in interphase nuclei after replication. This interpretation is supported by the observation that duplication of telomeric signals in interphase nuclei only occurs during the second half of the S-phase [65], while by this time, as shown here, almost half of telomeres have already replicated. It is clear that the ReD-FISH approach, although laborious and time consuming, has allowed to define in a more precise way the timings of replication of single telomeres in human cells.

One striking feature of the telomere replication pattern in human cells is the late replication timing of telomeres associated with satellite-like repeats, i.e. the short arm of the acrocentric chromosomes as well as 4qter and 10qter extremities. Our experiments using newly created tagged telomeres indicate that the presence of β-satellite sequences, which have high heterochromatinization potential and are late replicated when in their natural context [29], caused the nearby telomere to replicate significantly later than control telomeres. Strikingly, the presence of a single D4Z4 repeat, which is sufficient to increase the association of a telomere with the nuclear periphery, caused the nearby telomeres to replicate much later in the S-phase than the control telomeres and as late as the acrocentric telomeres in the same cell line. Both effects, peripheral nuclear localization and late replication, are no longer detected when multiple D4Z4 repeats are inserted next to the telomere, further supporting the connection between subnuclear localization and telomere replication timing.

The reason why a single D4Z4 is able to mediate the association of a chromosome extremity to the periphery, while multiple copies of this repeat are not, remains mysterious. However, this observation is in agreement with the fact that the presence of multiple copies of D4Z4 at other locations, such as 10q and acrocentric telomeres, is not sufficient to increase the association of these extremities with the nuclear periphery [54]–[56]. As discussed in a previous work [56], the explanation for this apparent paradox may rely on the function of a putative region centromeric to the D4Z4 repeats and only present on 4q extremities. Independently of the mechanism involved in this perinuclear association, our experiments clearly point to a tight relationship between the peripheral localization of a telomere and its late replication behavior. On the other hand, the effect of β-satellite sequences appeared to be independent of nuclear localization. It is theoretically possible that a biased genomic integration of such constructs could have placed the newly created telomeres in a context where replication is intrinsically delayed. However, as shown previously within the limit of resolution of multi-FISH analyses [56], the telomere seeding strategy used here does not lead to a biased distribution of telomere seeds in C33A cells, supporting the contention that the observed effects are directly connected to the presence of particular juxtatelomeric elements carried by the constructions.

In yeast, the well-documented association of telomeres with the nuclear envelope [66] appears to play important roles in telomere metabolism, including length regulation [67], silencing [68] and repair [69], [70]. In humans, telomeres are supposed to be randomly distributed within the nucleus [71], but there have been reported exceptions, such as the nuclear peripheral localization of 4q [54], [55] and the perinucleolar localization of telomeres on the short arms of acrocentrics [53]. Strikingly, we found that other non-D4Z4 associated chromosome extremities are also naturally localized at the nuclear periphery in unperturbed IMR90 cells, adding four more exceptions (2p, 3p, 6q and 12q) to the list of telomeres with preferential nuclear localizations. Remarkably, all these telomeres replicate late in the diploid fibroblasts examined.

Thus, our observations point to a relationship between telomere nuclear localization and telomere replication timing. Nuclear localization has been suggested to affect replication timing of other regions of the genome [51], [72], [73]. Also, recent studies have demonstrated that genome-wide interactions with the nuclear lamina implicate late replicated sequences [74]. Close examination of the subtelomeric chromosome specific sequences available for the extremities examined here (http://genome.ucsc.edu/cgi-bin/hgGateway) revealed that only 12q present a particular enrichment in LADs (lamina-associated domains) [74]. However, actual subtelomeric regions are most often not included in human genome sequence assemblies, either because they are poorly characterized or because their duplicated nature makes their chromosome assignment quite difficult. It is worth noting here that at least the last 120 kilobases of the subtelomeric region of the 6q chromosome are duplicated on other extremities, including 1p [48], whose telomere, contrary to that one on 6q, replicates early and is not associated with the nuclear periphery.

Our results also indicate that single telomere replication timing in human diploid fibroblasts is mostly determined by chromosome-specific features, perhaps at the level of large chromosome domains, as suggested recently [72]. Although telomere replication timings do not appear to be correlated with the replication timings of large cytogenetic bands, we did find a correlation between the mean replication timings for single telomeres and the timing of replication reported for the most distal chromosome-specific sequences present in a BAC-array [40]. This correlation, albeit weak (and only statistically significant when data from telomerized diploid fibroblasts were used, perhaps reflecting the fact that the BAC study was conducted in an EBV-transformed lymphocyte cell line [40]), suggests that telomere replication may be, at least partially, synchronized with chromosome-specific subtelomeric sequences.

Finally, our data conclusively show that telomere replication timing may also be influenced by the presence of relatively small telomere-associated sequences, such as β-satellite sequences or one repeat of the macrosatellite D4Z4, which also confers a peripheral nuclear localization to the chromosome end. It has been recently demonstrated that this D4Z4 sequence behaves both as an A-type lamin-, CTCF-dependent peripheral tethering element and as an insulator [56], [58]. It remains to be determined which of these two properties confer late replication.

Together, our study allowed an original and certainly informative glimpse into the mechanisms regulating telomere replication timing in human cells. Our results suggest that the links between replication timing and high-order genome organization, also observed in other organisms, may have been conserved throughout evolution [28], [41], [75], [76].

## Materials and Methods

The human cell lines used here include the fetal lung fibroblast IMR90 (46XX, obtained from ATCC) and the foreskin fibroblast HCA2 (46XY, obtained from James Smith, Baylor College). Both cell lines were immortalized by transduction of a pBABE-derived retrovirus carrying hTERT. IMR90+hTERT was also transduced with another retrovirus carrying TINF2. We also constructed derivatives of the well-known cervical carcinoma cell line C33A [58]. The different constructs carry a hygromycin resistance gene fused to the herpes simplex virus type 1 thymidine kinase suicide gene and an *eGFP* reporter gene, each driven by a CMV promoter (see Figure 6A for description of the constructs). A telomere seed is added downstream of the integrated sequences in order to create a *de novo* telomere after random integration followed by a telomeric fragmentation [57]. A single *D4Z4* repeat (black box) or 8 tandem copies of the repeat were cloned between *eGFP* and the telomere seed in T construct (T1X) as previously described [56]. At different loci, D4Z4 co-segregates with β-satellite sequences. A PCR-amplified fragment of 1.4 kb corresponding to the distal sequence of the 4q35 subtelomere and encompassing 4 β-satellite elements (Boussouar et al., manuscript in preparation) was also cloned in the T construct, either downstream of the *eGPF* reporter or downstream of the D4Z4 element. After transfection and telomeric fragmentation, the cells were grown and selected for antibiotic resistance in polyclonal batches. The location of newly created telomeres (90% of the cells in a given population) differs between cells [56].

For replication studies, all cell lines were synchronized by a double thymidin/aphidicholin block. Briefly, cells were incubated for 16 h in 2 mM thymidine, released in S after 3 washes of PBS pre-warmed to 37°C and 8 to 12 h later, depending on the cell line, treated again with 1µg/ml aphidicolin for 16 h prior to release in S after three washes in pre-warmed PBS.

### Cell cycle analysis

To precisely determine the length of the S-phase, cells were analyzed by FACs. Every hour after release from the aphidicolin block, cells were trypsinized, centrifuged, resuspended in 0.5 ml of PBS, fixed by drop-wise addition of 1.5ml ice-cold 100% ethanol and stored at 4°C. Subsequently, cells were centrifuged and resuspended in a staining solution containing 30 µg of propidium iodide and 200 µg of RNaseA per ml. Flow cytometry was performed using a Becton Dickinson FACSort flow cytometer. The data was analyzed using FlowJo software.

### BrdU/BrdC pulses and metaphase preparations

Replicative Detargeting (ReD) FISH is a modification of the CO-FISH procedure [34] and was performed as described previously [33] with some modifications. Briefly, after release from the aphidicolin block, 6 to 8 pulse-chase additions of BrdU/BrdC were made depending on the length of the S-phase. For each pulse, cells were incubated for 1 hour in the presence of 10 µM BrdU and 3.3 µM BrdC, then washed 3 times with pre-warmed PBS before new media was added. 7 to 10 hours after release from the aphidicolin block, cells were arrested in mitosis with 1.5 hour incubation in colcemid (0.1 µg/ml) before 40 min hypotonic shock in 0.8 g/L sodium citrate at 37°C and fixed in ethanol/acetic acid. Metaphase spreads were obtained by dropping suspensions of fixed cells onto clean glass slides and were rapidly used for hybridization. Spreads were denatured at 80°C for 4 min in the presence of a Cy3-(CCCTAA)_3_ PNA probe (Applied Biosystems, 50 nM in 70% formamide, 25 mM Tris pH 7.4) and incubated at room temperature for 2 hours. After washes and ethanol dehydration, the slides were put in contact for 2h with a second LNA probe 5′-(6-Fam)GGGtTAGGGttAgGGTTAGGgttAgGgttTAGGgTTA (6-Fam)-3′ - where small letters correspond to positions with locked nucleic acids - (Proligo-France, 10 mM in 50% formamide, 2xSSC) followed again by washes and ethanol dehydration. Preparations were mounted in Vectashield (Vector) with DAPI (1 µg/ml) and visualized with a Zeiss UV microscope equipped with appropriate excitation/emission filters for each color. Images were captured with a HQ-Coolsnap camera (Photometrics) using the IPlab software. When required, coordinates for all metaphases were recorded in order to retrieve them after a second (subtelomeric) FISH.

### Quantitative (Q-)FISH

The Q-FISH procedure was carried out exactly as described, using a Cy3-(CCCTAA)_3_ PNA probe [9]. Metaphase spreads, prepared the day before, were fixed with formaldehyde (Sigma, 3.7%) and digested with pepsin (Sigma, 1 mg/ml). Spreads were denatured at 80°C for 4 min in the presence of a Cy3-(CCCTAA)_3_ PNA probe (Applied Biosystems, 50 nM in 70% formamide, 25 mM Tris pH 7.4) and incubated at room temperature for 2 hours. After washes and ethanol dehydration, preparations were mounted in Vectashield (Vector) with DAPI (1 µg/ml) and visualized with a Zeiss UV microscope equipped with appropriate excitation/emission filters. Images were captured with a HQ-Coolsnap camera (Photometrics) using the IPlab software. When required, coordinates for all metaphases were recorded in order to retrieve them after a second (subtelomeric) FISH.

### Subtelomeric FISH

After CO-FISH or Q-FISH hybridizations, slides where washed 3 times in SSC 2X and dehydrated for subsequent subtelomeric FISH to distinguish homologues. Cosmids carrying subtelomeric regions, f7501 and ICRF10, were obtained from Barbara Trask (Human Genome Center, Lawrence Livermore National Laboratory) and Gilles Vergnaud (IGM, Orsay, France), respectively. Cosmid f7501 contains 36-kb portion of chromosome 19 including three members of the olfactory receptor (OR) family [49]. Cosmid ICRF10 carries minisatellite DNF92 (GenBank accession number Y13543) [46], [48]. Subtelomeric probes f7501 and ICRF10 correspond to two different segments of around 35 kb located close to the telomere tract on around 15 different chromosome extremities. The presence of one segment is exclusive of the other and both segments are typically associated with particular arrangements of other, more centromeric, segments. These probes were used for hybridization on metaphase preparations that had already been analyzed in either Q-FISH or ReDFISH experiments, thus allowing to distinguish between allelic chromosome extremities and to conduct allele-specific telomere fluorescence measurements (as described in [9]) or replication timing scorings (this paper).

One g of cosmid DNA was labeled with biotin-16-dUTP (Roche) using the Nick translation kit (Vysis) following manufacturer's instructions. For hybridization, 50 ng probe per slide was precipitated in the presence of 100 µg single-strand salmon sperm DNA and 20 µg COT-1 (Invitrogen), dissolved in 25 l hybridization mix (50% formamide, 10% dextran sulfate, SSC 2X) and pre-hybridized for 1 h at 37°C. Slides with metaphase spreads were treated with 0.1 g/ml RNase A in SSC 2X for 1 h at 37°C and washed three times in SSC 2X, 5 min each, prior to denaturation in 70% formamide/SSC 2X at 70°C for 2 min. Denaturated slides were dropped in ice-cold SSC 2X, dehydrated in a series of ice-cold ethanol baths, treated with Proteinase K (100 ng/ml in 20 mM Tris pH 7.4 and 2 mM CaCl_2_) for 8 min at 37°C, dehydrated again and hybridized over night at 37°C. The next day slides were washed three times, 3 min each in 50% formamide/SSC 2X, five times, 2 min each in SSC 2X, and once in BN (0,1 M sodium bicharbonate, 0,05% NP-40) all at 45°C. Slides were blocked with 5% milk in BN for 15 min. Biotinylated probes were detected with three layers of antibodies, each 30 min at 37°C, as follows: fluorescein avidin D (Vector A-2001, 1/400), biotinylated anti-avidin (Vector BA-0300, 1/100) and fluorescein avidin D, all diluted in blocking buffer. After each antibody layer three 2-min washes in BN at 42°C were done. Slides were mounted in Vectashield (Vector) with 0.2 g/ml DAPI.

To detect newly seeded telomeres in the C33A cell derivatives after ReD-FISH, a labeled pCMV vector was used as a probe and labeled with the DIG-Nick Translation Kit (Roche Diagnostics). All probes were denatured at 80±1°C for 5 minutes before hybridization. Conditions for slide preparation, hybridization and immunodetection have been described [56]. For detection, we used mouse anti-DIG antibodies (Roche Diagnostics), diluted 1/200, followed by incubation with secondary donkey antibodies coupled with ALEXA 488 fluorochrome, directed against this epitope and diluted 1/500 (Molecular Probes). Chromosomes were counterstained with DAPI antifade (0.125 µg/ml) (Cytocell).

Metaphases were retrieved thanks to the recorded coordinates and original images were annotated.

### ReDFISH analysis

For each pulse, 30–50 metaphases were captured and analyzed. For each metaphase, a karyotype was carried out and chromosome ends with detargeted telomeres were identified. For each BrdU/C pulse, the average percentage of detargeted telomeres in the population of metaphases was calculated for each pair of chromosome ends. The addition of these percentages from all 6 BrdU pulses spanning the entire S phase was adjusted to 100%. The mean replication timing of single telomeres was calculated as follows: mrt = [Y]/f, where Y corresponds to the pulse (0.5, 1.5, …) and f is the percentage of telomeres seen replicating during that pulse. For the graphic representation of replication timings of individual telomeres, percentages of replication were grouped in early S (pulses 1+2), mid-S (pulses 3+4) and late S (pulses 5+6) for each telomere and telomeres were ordered according to their mrt.

### Telomere restriction fragments analysis (TRF)

Ten µg of genomic DNA was digested overnight with *Hinf*I and *Rsa*I enzymes, 50U each, and restriction fragments were separated through a pulsed-field electrophoresis agarose gel (1% in TBE 0.5X) in a CHEF apparatus (BioRad) set to 200 V for 15 h with a pulse ramp between 0.2 and 13 s. After staining with ethidium bromide, DNA was nicked by a UV-crosslinker (Stratagene) at 180,000 J/cm^2^, denatured, and transferred by capillary alkaline transfer onto Biodyne B Nylon membrane (Pall) for hybridization with a radioactively labeled TAA(CCCTAA)_4_ oligonucleotide. Signals were detected in a phosphorimager apparatus.

### 3D immuno-FISH

To determine the localization of telomeres in the nucleus, PAC clones recognizing the terminal region of chromosome arms 1p, 2p, 3p, 4q, 5p, 6q, 12p, 12q, 1 and 17q [77] were labeled with the DIG-Nick Translation Kit (Roche Diagnostics). All probes were denatured at 80±1°C for 5 minutes before hybridization. Conditions for slides preparation, hybridization and immunodetection have been described [56].

For detection, we used mouse anti-DIG antibodies (Roche Diagnostics) and goat anti-Lamin B antibodies (M-20, Santa-Cruz), diluted 1/50, followed by incubation with secondary donkey antibodies coupled with different ALEXA fluorochromes, directed against these epitopes and diluted 1/300 (Molecular Probes). Nuclei were counterstained with DAPI antifade (0.125 µg/ml) (Cytocell).

Images were acquired with the confocal scanning laser system, LSM510, from Zeiss (Germany). A 63× Plan-APOCHROMAT, oil immersion, NA 1.40 objective (Zeiss) was used to record optical sections at intervals of 0.48µm. The pinhole was set the closest to 1 Airy with optical slices in all wavelengths with identical thickness (0.8µm). Images were averaged 4 times to improve the signal to noise ratio. Generated .lsm files had a voxel size of 0.1µm×0.1µm×0.48µm and were processed through the Imaris software (Bitplane AG). After 3D analysis, where at least 50 nuclei were examined, data sets are presented as the distribution of FISH signals between three concentric zones of equal volume or as the mean ratio between two volumes.

### Statistical analyses

The R package was used for comparisons using Fisher exact tests and Spearman's rank correlation coefficient calculations. For multiple comparisons, corrections for significance thresholds were applied depending on the number of comparisons actually carried out (p<0.05/k; Bonferroni): k = 20 for Fisher exact test comparisons using diploid fibroblast data and k = 15 for comparisons using C33A data.

## Supporting Information

Figure S1 The mean replication timings of single telomeres are weakly correlated to the mean replication timings of the most distal chromosome-specific sequences. One-to-one coefficient correlation analyses were conducted taking, on one hand, the mean replication timings for single telomeres in the three different cell lines we examined and, on the other hand, the reported mean replication timing values for chromosome sequences carried by the most distal BAC on the same extremities analyzed in the paper by Woodfine et al. [40]. Values for telomere-specific mean replication timing increase with S-phase progression while values for BAC-specific mean replication timing decrease with S-phase progression, and therefore a negative correlation will indicate that there is a trend to replicate synchronously. As shown, this trend is only statistically significant for comparisons between telomerized fibroblast cell lines (IMR90+TT and HCA2+T) and the lymphoblastoid cell line (LCL).
(0.71 MB EPS)

Figure S2 Telomeres on the long arm of sex chromosomes tend to replicate late. (A) In the female cell line IMR90, both Xp telomeres appear to replicate synchronously near the middle S-phase while replication of telomeres on the long arms shows a biphasic distribution. It is hypothesized that the peak of late replication corresponds to the q telomere of iX, but distinction between active and inactivated X chromosomes is not feasible under our experimental conditions (see Results). (B) In the male cell line HCA2+T, both Xp and Xq telomeres appear to have a peak of replication near mid-S, but the mrt of Xq telomeres occurs significantly later than Xp. On the other hand, peaks of replication (and mean replication timings) of Y telomeres show one hour gap, with q telomeres replicating later in S. Differences between the mrt of Xp and Yp and between the mrt of Xq and Yq are not statistically significant (significance threshold p<0.0025). Vertical blue and green lines indicate the mean replication timing for each telomere (overall mean replication timing in gray).
(0.57 MB EPS)
